# The effects of elevated CO_2_ (0.5%) on chloroplasts in the tetraploid black locust (*Robinia pseudoacacia* L.)

**DOI:** 10.1002/ece3.3545

**Published:** 2017-11-01

**Authors:** Yuan Cao, Mingquan Jiang, Fuling Xu, Shuo Liu, Fanjuan Meng

**Affiliations:** ^1^ College of Life Science Northeast Forestry University Harbin China; ^2^ Jilin Province Product Quality Supervision and Inspection Institute Changchun China

**Keywords:** chloroplast, diploid, elevated CO_2_, photosynthesis, tetraploid, tetraploid black locust (*Robinia pseudoacacia* L.)

## Abstract

Some ploidy plants demonstrate environmental stress tolerance. Tetraploid (4×) black locust (*Robinia pseudoacacia* L.) exhibits less chlorosis in response to high CO_2_ than do the corresponding diploid (2×) plants of this species. We investigated the plant growth, anatomy, photosynthetic ability, chlorophyll (chl) fluorescence, and antioxidase activities in 2× and 4× black locusts cultivated under high CO_2_ (0.5%). Elevated CO_2_ (0.5%) induced a global decrease in the contents of total chl, chl a, and chl b in 2× leaves, while few changes were found in the chl content of 4× leaves. Analyses of the chl fluorescence intensity, maximum quantum yield of photosystem II (PSII) photochemistry (*Fv/Fm*), K‐step (*V*_k_), and J‐step (*V*_J_) revealed that 0.5% CO_2_ had a negative effect on the photosynthetic capacity and growth of the 2× plants, especially the performance of PSII. In contrast, there was no significant effect of high CO_2_ on the growth of the 4× plants. These analyses indicate that the decreased inhibition of the growth of 4× plants by high CO_2_ (0.5%) may be attributed to an improved photosynthetic capacity, pigment content, and ultrastructure of the chloroplast compared to 2× plants.

## 1 INTRODUCTION

Over the last two centuries, the global atmospheric carbon dioxide concentration [CO_2_] has increased faster than predicted and will double within the next hundred years (Meehl et al., 2007; Peters et al., 2011). Elevated CO_2_ (EC) is largely responsible for plant growth and yield. Generally, an increase in CO_2_ concentration increases photosynthesis, stimulates growth, and improves yield (Reef et al., 2015; Stiling et al., 2013; Taub, 2008). Furthermore, EC influences the morphology, respiration, and proliferation of plants (Barnes, Ollerenshaw, & Whitfield, 1995; Benlloch‐Gonzalez, Berger, Bramley, Rebetzke, & Palta, 2014; Gifford, 1995; Roden and Ball 1996; Wang et al., 2012). For plants, these positive effects of EC may be due to higher stomatal conductance and water‐use efficiency (WUE) (Onoda, Hirose, & Hikosaka, 2009). Previous studies have mostly focused on the responses of crops such as wheat (Gifford, 1995), cotton (Hu et al., 2013), and soybean (Bunce, 2014) or non‐fast‐growing tree species, including oak (Stover, Day, Ke, & Hinkle, 2010) and pine (Phillips, Finzi, & Bernhardt, 2011), to EC conditions. Or some metabolic networks have been analyzed due to their biotechnological and basic science importance: the photosynthetic carbon metabolism in a general leaf, the *Rhodobacter spheroides bacterium*, and the *Chlamydomonas reinhardtii alga* (Carapezza et al., 2013). However, for polyploidy plants, the effects of elevated CO_2_ on photosynthesis and growth are not clear.

Tetraploid (4×) black locust (*Robinia pseudoacacia* L.) (TBL), which is native to Korea, not only is a preferred tree species in timber forests because of its rapid growth and good wood texture but also can be used as a superior feed for domestic fowl and livestock because its fleshy leaves are rich in vitamins and minerals (Meng, Pang, Huang, Liu, & Wang, 2012; Wang, Wang, Liu, & Meng, 2013). Moreover, TBL has a wide range of adaptability to adverse abiotic and biotic environmental conditions (Li et al., 2009; Podda et al., 2013; Zhang & Forde, 2000). Therefore, TBL has higher economic and ecological value due to these properties.

At present, polyploidy induction has become an important method to gain insight into physiological mechanisms of plants in the response to environmental stress (Comai, 2005; Woode et al., 2009). In fact, many polyploidy plants have a superior tolerance to environmental stresses compared to their corresponding diploid (2×) plants (Huang et al. 2002; Zhang, Hu, & Yao, 2010). Chloroplasts, one of the primary organelles, are more sensitive to various environmental stresses than other organelles because photosynthesis and other biochemical and biophysical processes occur within these structures (Barry & John, 1981; Wang et al., 2013). However, the mechanisms by which EC affects chloroplasts are not completely understood. In particular, few studies have explored the special response mechanisms of chloroplasts from polyploidy woody plants to specific EC conditions. Indeed, changes in the response to EC of the chloroplasts from polyploidy plants could have a significant ecological impact.

The aim of this study was to determine the effects of a particular EC concentration (0.5%) on two black locust species (2× and 4×). Based on the response of TBL to other abiotic stresses (Meng et al., 2012; Wang et al., 2013), we proposed the following hypotheses: (1) TBL (fast‐growing trees) may adapt better to EC conditions compared to its 2× counterpart, and (2) TBL may adapt to an EC environment via adjustments at the chloroplast level. Accordingly, to determine the physiological and biochemical responses of the 2× and 4× plants to a particular EC condition (0.5%), we measured and observed the morphology, anatomy, photosynthetic ability, antioxidase activities, and ultrastructure and other parameters of the chloroplasts.

## 2 MATERIALS AND METHODS

### 2.1 Plant growth

All of the materials were introduced into China directly from Korea by Beijing Forestry University. The 2× and 4× black locust (*Robinia pseudoacacia* L.) seedlings were from same germplasm and had the same genetic base (Wang et al., 2013). In June 2014, 30 plants (1 year old) from among the 2× and 4× species were selected for size uniformity and were grown in plastic pots (10 cm in diameter and 10 cm in depth) filled with a 2:1 (v/v) mixture of soil and sand. This experiment was carried out in a high‐performance CO_2_‐controlled growth chamber (Huanghua Faithful Instrument Co., Ltd., Hebei, China) under 0.5% CO_2_ concentrations as treated with a light/dark cycle of 16 hr/8 hr, at temperatures of 30/25°C, 50/60% humidity, and 250 μmol photons m^−2^ s^−1^ light. The seedlings were harvested and measured followed by treatment as control (day 0) and then harvested and measured again at 6 days after the treatment (day 6).

### 2.2 Determination of leaf morphology, leaf nitrogen (N), and carbon isotope composition (δ^13^C)

On day harvested, the leaves morphology was photographed. For the determination of nitrate concentrations and carbon isotope composition (δ^13^C), the leaves were immediately weighed and dried at 80°C for 48 hr to a constant weight. Nitrate concentrations were determined according to the method of Yin et al. (2012). δ^13^C was analyzed using a mass spectrometer (Finnegan MAT Delta‐E, Germany) (Li et al., 2009).

### 2.3 Determination of chlorophyll pigment

Total chlorophyll, chlorophyll *a*, and chlorophyll *b* contents were estimated according to the methods of Meng et al. (2012). 300 mg of fresh leaves was ground in 80% cold acetone and centrifuged at 12,000 *g* for 20 min. Then, the supernatant was collected and fixed in 10 ml of 80% acetone and detected at 470, 645, and 663 nm.

### 2.4 Determination of photosynthesis and chlorophyll fluorescence

Net photosynthetic rates (Pn), stomata conductance (Gs), intercellular CO_2_ (Ci) of leaves were measured from 09:00 to 11:30 in the morning using a LI‐6400 photosynthesis system (Li‐Cor, LI‐COR Biosciences, Lincoln, NE, USA). The ambient CO_2_ concentration, leaf temperature, humidity, and leaf‐to‐air vapor pressure deficit were 390 ± 10 μmol/L, 25°C, 50%, 1–1.3 kPa. Chlorophyll fluorescence parameters were recorded using a Handy PEA (Hansatech Instruments, Ltd., King's Lynn Norfolk, UK) (Meng et al., 2012). Measurements were repeated at least five times for each treatment.

### 2.5 Isolation of chloroplasts

To isolate and purify chloroplasts from the leaves, we followed protocols described by Yin et al. (2011) with minor modifications. All of the steps were performed at 4°C. In total, 30 g of leaves was harvested and ground in 200 ml of isolation buffer I containing 50 mmol/L HEPES (hydroxyethyl piperazine ethanesulfonic acid)/KOH (pH 7.5), 5 mmol/L hexanoic acid, 0.3% bovine serum albumin (BSA) (w/v), 0.3 mol/L sucrose, 10 mmol/L β‐mercaptoethanol, 20 mmol/L ethylenediaminetetraacetic acid (EDTA), 30 mmol/L Na‐ascorbate, and 1% (w/v) polyvinylpyrrolidone (PVP). Then, the homogenate was filtered through six layers of mesh nylon cloth (40 × 40 μm) and centrifuged at 4,000 *g* for 10 min. The supernatant was centrifuged at 20,000 *g* for 10 min. The precipitate was carefully suspended in buffer II containing 20 mmol/L HEPES/KOH (pH 7.5), 330 mmol/L sorbitol, 10 mmol/L NaCl, 2 mmol/L EDTA, and 5 mmol/L Na‐ascorbate and washed twice. Subsequently, the resuspended chloroplasts were loaded onto a Percoll gradient consisting of a 6:6:6:3 ratio, top to bottom, of 10%, 40%, 70% and 90% Percoll. The mixture was centrifuged for 0.5 hR at 40,000 *g*, and the chloroplasts were present between the 40% and 70% interface. Then, the intact chloroplasts were collected, washed, and centrifuged at 10,000 *g* for 15 min in buffer II medium.

### 2.6 Measurement of the lipid peroxides and hydrogen peroxide of chloroplasts

Lipid peroxides were estimated using a modified method of Janik‐Papis et al. (2009). In total, 0.5 ml of chloroplast supernatant was added to a test tube containing 2 ml of a mixture of 20% TCA (Trichloroacetic acid), 0.01% butylated hydroxytoluene, and 0.6% thiobarbituric acid. The mixture was heated in boiling water for 30 min and then quickly cooled on ice. After centrifugation at 12,000 *g* for 10 min, the absorbance of the supernatant was determined at 450, 532 and 600 nm. Hydrogen peroxide (H_2_O_2_) was detected spectrophotometrically according to the method of Sergiev et al. (1997). A volume of 0.5 ml of chloroplast supernatant was homogenized in 0.1% TCA in an ice bath. After centrifugation at 12,000 *g* for 10 min, 0.5 ml of the extracted solution was mixed with 0.5 ml of potassium phosphate buffer (pH 7.5) and 1 ml of potassium iodide (1 mol/L). The absorbance of the supernatant was measured at 390 nm, and the concentration of H_2_O_2_ was obtained using a standard curve.

### 2.7 Measurement of the enzymatic activities of chloroplasts

Superoxide dismutase (SOD, EC1.15.1.1) activity was measured following the method of Beauchamp and Fridovich (1971). The reaction mixture contained 20 μl of enzyme extract, 50 mmol/L sodium phosphate buffer (pH 7.8), 100 μmol/L EDTA, and 10 mmol/L pyrogallol. Enzymatic activity was detected spectrophotometrically at 420 nm. Glutathione reductase (GR, EC1.6.4.2) activity was determined via nicotinamide adenine dinucleotide phosphate (NADPH) oxidation at 340 nm. The reaction mixture contained 10 μl of enzyme extract, 100 mmol/L potassium phosphate buffer (pH 7.8), 0.2 mmol/L NADPH, 2 mmol/L EDTA, and 0.5 mmol/L glutathione. The reaction was initiated by adding NADPH at 25°C (Carlberg & Mannervik, 1975). Peroxidase (POD, EC.1.11.1.7) activity was measured according to the method of Nickel and Ba (1969). Ascorbate peroxidase (APX, EC1.11.1.11) activity was assayed by the method of Nakano and Asada (1981). The reaction mixture contained 50 mmol/L sodium phosphate buffer (pH 7) including 0.2 mmol/L EDTA, 0.5 mmol/L ascorbic acid, and 50 mg of BSA. The reaction was started by adding H_2_O_2_ at a final concentration of 0.1 mmol/L.

## 3 RESULTS

### 3.1 Changes in morphology and MDA and H_2_O_2_ levels

**Figure 1:**
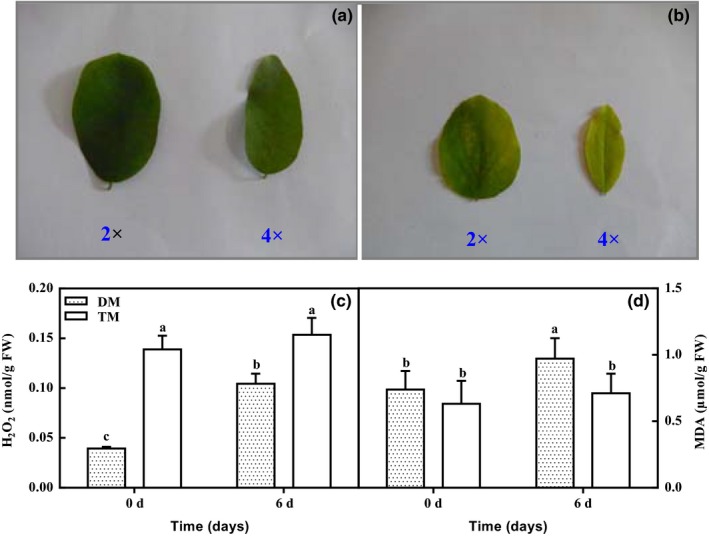
Morphology (a and b), H_2_O_2_ (c), and MDA (d) contents in chloroplasts of leaves in 2× and 4× at 0 and 6 days, respectively. (a) Morphology of leaves of 2× (diploid black locust) and 4× (tetraploid black locust) at 0 day; (b) morphology of leaves of 2× and 4× at 6 days; (c) H_2_O_2_ content of leaves of 2× and 4× at 0 and 6 days, respectively; (d) MDA content of leaves of 2× and 4× at 0 and 6 days, respectively; DM, 2×; TM, 4×

High EC led to chlorosis in the leaves of both 2× and 4× seedlings compared with their corresponding controls (Figure 1a). Furthermore, the 2× leaves exhibited more obvious and severe chlorosis than the leaves from the 4× seedlings (Figure 1b). In addition, H_2_O_2_ and MDA (malondialdehyde) accumulated significantly in the 2× plants under 0.5% CO_2_ conditions. Conversely, there were no differences in the H_2_O_2_ and MDA contents of the 4× plants between the control and treated (0.5%) samples (Figure 1c,d).

### 3.2 Chlorophyll content, N content, and carbon isotope composition (δ^13^C)

The contents of total chlorophyll (chl), chl *a* and chl *b* of 2× and 4× plants are presented in Table 1. In the 2× species, statistically significantly lower contents of total chl (52.86%), chl *a* (51.11%), and chl *b* (57.14%) were detected after 0.5% CO_2_ treatment, whereas no significant changes were found in total chl, chl *a,* and chl *b* of the 4× plants under 0.5% CO_2_ conditions. In contrast, 0.5% CO_2_ did not significantly affect the carbon isotope composition (δ^13^C) or N content of the 2× and 4× plants.

**Table 1:** Chlorophyll *a,* chlorophyll *b*, total chlorophyll contents, N content, and carbon isotope composition (δ^13^C) in leaves of 2× and 4× affected by high CO_2_ condition

		Chlorophyll *a* (mg/g)	Chlorophyll *b* (mg/g)	Total chlorophyll (mg/g)	δ^13^C (‰)	N (g/kg)
2×	Control	0.765 ± 0.057^a^	0.336 ± 0.029^a^	1.101 ± 0.083^a^	−29.28 ± 0.17^b^	12.10 ± 0.28^a^
	Treatment	0.374 ± 0.058^b^	0.144 ± 0.031^b^	0.519 ± 0.088^b^	−29.05 ± 0.10^b^	12.80 ± 0.18^a^
4×	Control	0.807 ± 0.040^a^	0.337 ± 0.033^a^	1.144 ± 0.072^a^	−33.08 ± 0.06^a^	11.95 ± 0.23^a^
	Treatment	0.786 ± 0.113^a^	0.323 ± 0.053^a^	1.109 ± 0.166^a^	−32.37 ± 0.16^a^	12.08 ± 0.24^a^

Different small letters mean significant differences in the same parameters (*p *<* *.05).

### 3.3 Chloroplast ultrastructure

**Figure 2:**
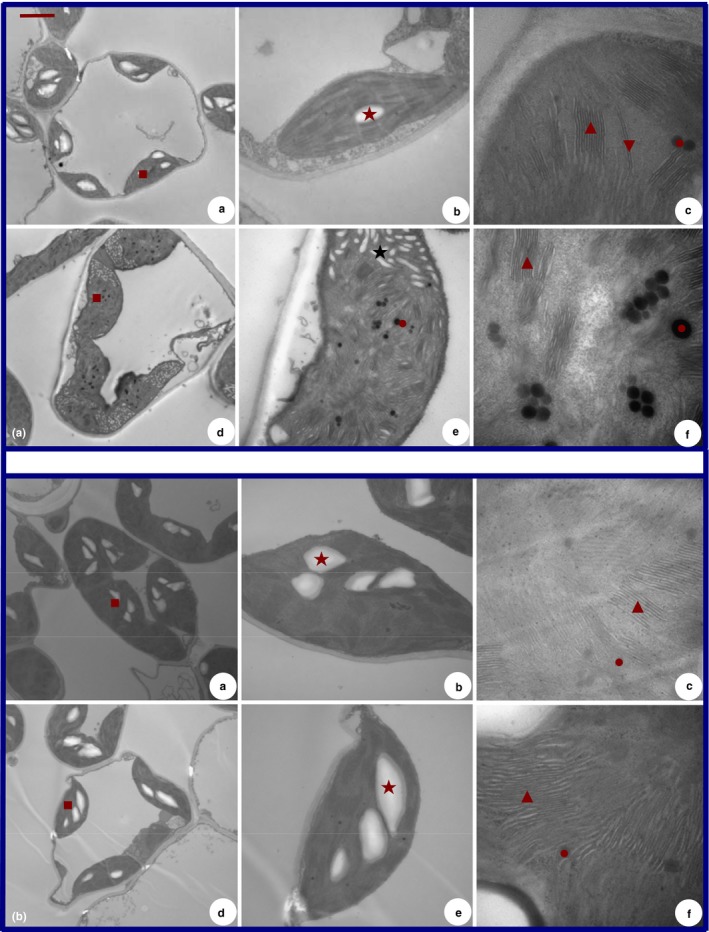
Transmission electron micrographs of chloroplasts in 2× (a) and 4× (b) at 0 (up) and 6 (down) days, respectively. 2×, 4×, a and d (bar = 0.25 μm), arrangement of chloroplast; b and e (bar = 0.5 μm), ultrastructure of chloroplast; c and f (bar = 100 nm), grana thylakoids of chloroplast. Five‐pointed star (★), starch grains; square (■), chloroplast; up‐triangle (▲), grana; down‐triangle (▼), thylakoids; ball (●), liposomes

The ultrastructures of the 2× and 4× species are depicted in Figure 2. The ultrastructure and arrangement of the chloroplasts of both the 2× and 4× seedlings were normal under control conditions, but injuries were apparent in the 2× plant under 0.5% CO_2_ conditions (Figure 2A). Under control conditions, grana (Gr) of both the 2× and 4× seedlings were well developed and highly stacked with normal thylakoids (Figure 2). In the 2× plants, 0.5% CO_2_ induced a disturbance of the chloroplast structures. Swollen, instead of smooth and spindly, chloroplasts and marked swelling of the Gr and an incompact structure of thylakoids in the chloroplasts were found. The effects of starch and osmiophilic globule accumulation were also observed relative to controls (Figure 2A). In contrast, the 4× plants maintained structural integrity of the chloroplasts compared with those of the 2× plants after 0.5% treatment (Figure 2B). An important difference between diploid and tetraploid chloroplasts is the amount of osmiophilic bodies‐ in the diploid. About 0.5% CO_2_ induced increased numbers of osmiophilic globules in 2× plants (Figure 2A, e and f).

### 3.4 Measurement of gas exchanges

The photosynthetic rate (Pn), stomatal conductance (Gs), transpiration rate (Tr), and intercellular CO_2_ (Ci) in the 2× and 4× leaves decreased after exposure to 0.5% CO_2_ (Table 2). However, the decreases in the 2× leaves were much greater than those in the 4× leaves. Notably, the Pn and Ci in the 2× leaves decreased dramatically after high CO_2_ treatment.

**Table 2:** Gas exchange and chlorophyll fluorescence in leaves of 2× and 4× as affected by 0.5% CO_2_

Species	Treatment (days)	Pn (μmol m^−2^ s^−1^)	Gs (mmol m^−2 ^s^−1^)	Ci (μmol CO_2_ mol^−1^)	Tr (mmol m^−2 ^s^−1^)	NPQ
2×	0	7.21 ± 0.85^a^	0.26 ± 0.06^a^	236.81 ± 35.6^a^	2.14 ± 0.70^a^	0.72 ± 0.14^b^
	6	2.20 ± 0.41^b^	0.14 ± 0.01^b^	73.52 ± 47.92^c^	0.30 ± 0.06^b^	1.26 ± 0.03^a^
4×	0	9.88 ± 0.41^a^	0.30 ± 0.07^a^	247.36 ± 72.11^a^	2.49 ± 0.06^a^	1.35 ± 0.29^b^
	6	4.95 ± 0.81^b^	0.21 ± 0.05^b^	150.55 ± 22.92^b^	1.10 ± 0.21^b^	2.02 ± 0.04^a^

Pn, photosynthesis rate; Gs, stomatal conductance; Tr, transpiration rate; Fv/Fm, maximal efficiency of PS II; NPQ, nonphotochemical quenching. Data are presented as the mean ± *SD*. Different small letters mean significant differences in the same parameters (*p *<* *.05).

### 3.5 Measurement of chl fluorescence

**Figure 3:**
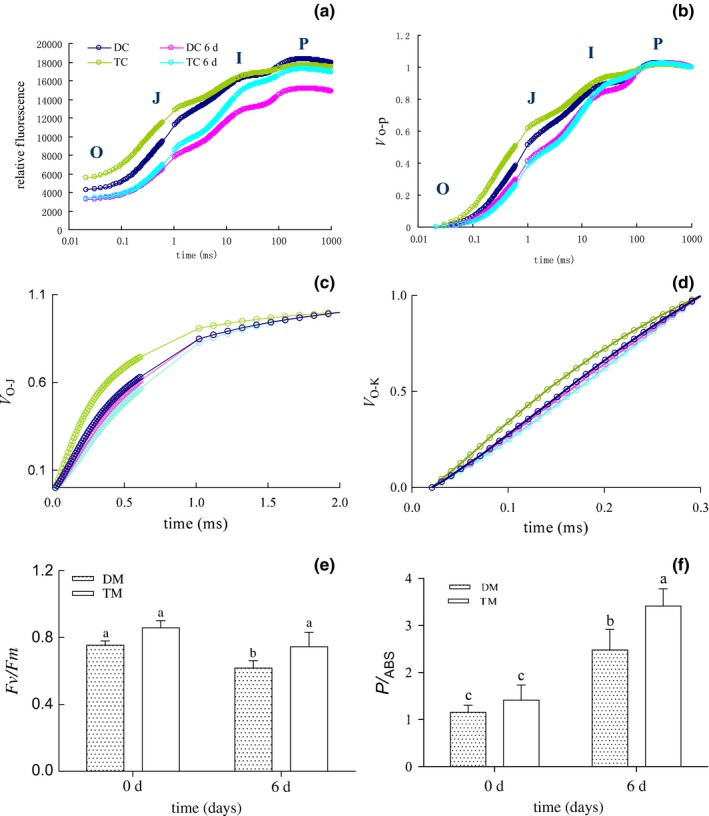
Chlorophyll fluorescence including relative fluorescence (a), *V*_o‐P_ (b), *V*_o‐J_ (c), *V*_o‐k_ (d), *Fv/Fm* (e), and *PI*_ABS_ (f) in leaves of 2× and 4× at 0 and 6 days, respectively. 2×, diploid black locust; 4×, tetraploid black locust; DC, 2× at 0 day; TC, 4× at 0 day; DC6d, 2× after 0.5% CO_2_ treatment of 6 days; TC6d, 4× after 0.5% CO_2_ treatment of 6 days; DM, 2×; TM, 4×

The 2× and 4× leaves exhibited a typical O‐J‐I‐P transient chl fluorescence upon exposure to 0.5% CO_2_ conditions and controls (Figure 3a). However, 0.5% CO_2_ lowered the fluorescence intensity, especially in I to P step of the 2× plants; thus, the shape of the O‐J‐I‐P transient was altered (Figure 3a). In addition, according to the *V*o‐p, the *V*_J_ (J‐step) (which occurred at approximately 2 ms) was decreased, and there was no difference in the *V*_I_ between the 2× and 4× plants after 0.5% CO_2_ treatment (Figure 3b). The values of *V*_k_ (K‐step) and *V*_J_ in the O‐J phase of the 4× samples were decreased after 0.5% CO_2_ treatment (Figure 3c). However, the 2× samples displayed little change in the values of *V*_k_ and *V*_J_ in the O‐J phase (Figure 3d). Furthermore, the maximum quantum yield of the PSII photochemistry (Fv/Fm) was reduced in the high‐CO_2_‐treated leaves of 2× plants (Figure 3e). The photosynthetic performance index (PI_ABS_) of both the 2× and 4× species increased after 0.5% CO_2_ treatment, while that of the 4× was higher compared with that of the 2× species (Figure 3f).

### 3.6 SOD, GR, POD, and APX activities

**Figure 4:**
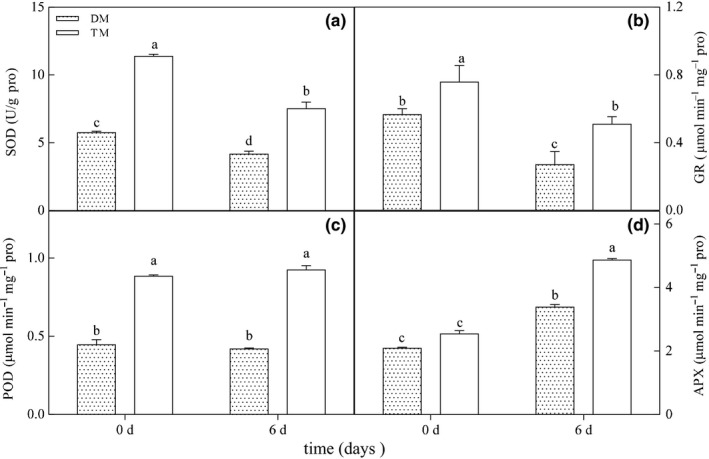
SOD (a), GR (b), POD (c), and APX (d) activities of chloroplasts in leaves of 2× and 4× at 0 and 6 days. 2×, diploid black locust; 4×, tetraploid black locust; DM, 2×; TM, 4×

The application of 5% CO_2_ decreased the activities of SOD, GR, and POD in the 2× plants by 27.49%, 52.21%, and 6.05%, respectively, while the APX activity exhibited a significant increase (38.38%) (Figure 4). However, in the 4× plants, the SOD and GR activities were marginally decreased, while the APX activity was greatly stimulated by 0.5% CO_2_ (Figure 4). In addition, the SOD, GR, POD, and APX activities in the 4× plants were much higher than those in the 2× plants both in control and treatment.

## 4 DISCUSSION

Chloroplasts are one of the vital organelles in plant cells because photosynthesis occurs in these structures. Many plant species display ultrastructural damage from environmental stress (Liu, Zhang, Qi, & Li, 2014). Accordingly, this damage to the chloroplast may lead to a significant decrease in photosynthesis (Zhang et al., 2010). This decrease in photosynthesis may result from rearrangements of the light‐harvesting complex I and light‐harvesting complex II (Garstka et al., 2005). In the current study, 0.5% CO_2_ induced a disturbance in the chloroplast structure by increasing the number of starch and osmiophilic globules (Figure 2). In other words, high CO_2_ conditions inhibit the growth of 2× plants due to a decrease in the Pn, reduction in the pigment content, destruction of the ultrastructure of the chloroplast. In contrast, there was no significant effect of high CO_2_ on the growth of the 4× plants. These results are consistent with results of previous studies (Jiang, Yang, & Zhang, 2007; Velikova et al., 2009). For example, potato plants accumulated more starch under long‐term CO_2_ enrichment (Sun, Li, & Liu, 2011). Hao et al. (2013) also reported that the accumulated starch within chloroplasts can cause a decline in photosynthesis with CO_2_ enrichment. This accumulation of starch grains may result in the arrangement of Gr thylakoids. Accordingly, such an alteration may lead to an inhibition of light energy absorption and a decrease in photosynthesis. Thus, 0.5% CO_2_ can affect a vital function in chloroplast structure regulation. However, the physiological, molecular, and biochemical mechanisms of starch accumulation and subsequent photosynthesis inhibition under high CO_2_ conditions remain unclear and require further investigation.

Both mineral nitrogen (N) and CO_2_ are involved in plant growth (Peterson et al., 1998). Our present study suggested that 0.5% CO_2_ increased the leaf N content of the 2× and 4× seedlings by 0.06% and 0.01%, respectively; however, this effect was not statistically significant (Table 1). This finding led us to conclude that elevated CO_2_ does not have a significant effect on N content. In fact, N is also a part of chl (Yin et al., 2012). However, elevated CO_2_ induced a decrease in chl *a*, chl *b,* and total chl of the 2× plants by approximately 50%, which could partly explain the decrease in the Pn (Table 2). Thus, the decrease in chl depends not on the effects of the N content but on other factors.

For plants, δ^13^C is chiefly controlled by the photosynthetic pathway (Lomax, Knight, & Lake, 2012). Farquhar, O'Leary, and Berry (1982) reported that the Tr can be estimated by measuring the δ^13^C in leaves during photosynthesis. Similar results have also been obtained in some legume crops, such as bean, cowpea, groundnut, and soybean (Kashiwagi et al. 2006). Therefore, there is a close relationship between the δ^13^C and Tr. However, no significant correlation was observed between the δ^13^C and Tr when the 2× and 4× plants were grown under 0.5% CO_2_ conditions (Tables 1 and 2). A similar result has been reported in sunflower (Virgona, Hubick, Rawson, Farquhar, & Downes, 1990). This indicated that the δ^13^C discrimination was limited in describing the Tr under elevated CO_2_ conditions. Alternatively, our results on black locust may indicate that the differences in the Tr are attributed to changes in Gs rather than by changes in mesophyll efficiency. These findings were extended, indicating that elevated CO_2_ negatively affects the Pn or Tr at N and δ^13^C levels.

Generally, an OJIP curve reflects the photochemical state in PSII (Tóth, Schansker, Garab, & Strasser, 2007). It is accepted that the shape of the initial fluorescence rise is reflected by the energetic connectivity or probability of exciton exchange between the PSII units (Yordanov et al., 2008). In higher plants, the OJIP curve possesses a sigmoidal shape under optimal conditions and is sensitive to various abiotic stresses (Lu, Qiu, Wang, & Zhang, 2003; Pierangelini, Stojkovic, Orr, & Beardall, 2014; Zhang et al., 2011). In our experiment, a decreased rate of the J‐step and I‐step was found in the 2× and 4× species after 0.5% CO_2_ treatment (Figure 3a,b). One may speculate that this effect could be related to the low absorption cross section of PSII and higher segregation of the photosystems, thereby reducing energetic connectivity between individual PSII units (Govindjee, 1995; Schansker, Tóth, & Strasser, 2005). The OJIP curves of the CO_2_‐treated 2× and 4× plants declined significantly (especially for the 2× sample), which indicated that an impairment of electron flow from PSII to the PQ pool occurred (Antal et al., 2006). In addition, a prominent decline in the P point was found in the 4× plant after 0.5% CO_2_ treatment, which may be due to the influence of nonphotochemical quenching (Antal et al., 2009).

To further characterize the function of the photosynthetic apparatus in response to the strain under the special EC condition (0.5% CO_2_), we also analyzed selected parameters derived from the OJIP curves. Generally, an increased rate of *V*k in the O‐J phase is a convenient indicator of the degree of damage of the oxygen‐evolving complex (OEC) activity (Pospíšil & Dau, 2000). The OEC in the membrane‐bound protein complex PSII catalyzes the water oxidation reaction that takes place in oxygenic photosynthetic organisms (Carina et al., 2013). In this study, the Vk value in the O‐J phase in both the 2× and 4× plants tended to decrease under elevated CO_2_ (0.5%) compared to the 2× plants (Figure 3c). The magnitude of this drop was higher in the 4× than in the 2× plants, indicating that the special CO_2_ may induce OEC activity to protect PSII. However, these changes were not confirmed by the structural changes of thylakoids in the 2× chloroplasts (Figure 2) because the inhibition of the OEC activity can lead to lipid peroxidation damage, an increase in membrane permeability, and the release of more liposomes (Takahiro, Mitsue, & Yasusi, 2004). Generally, the degradation of thylakoids can result in the formation of liposomes (Ma, Zhang, Wang, Song, & Zhang, 2013). Therefore, in the 2× plants, an incompact structure of thylakoids was found in the chloroplasts with more liposomes compared with the 4× chloroplasts, which suggests that the damage to the OEC in the 2× plants by 0.5% CO_2_ treatment was not more significant than that in the 4× plants (Figure 2). We also noted that the *L* value (*V*_L_, 0.15 ms) in the O‐K phase of the 4× sample was decreased after 0.5% CO_2_ treatment; however, this value only changed a little (Figure 3d). In general, the absorption, transmission, and transformation of light energy are carried out in the thylakoid membranes (Chow, Haehnel, & Anderson, 2006). The increase of the *V*_L_ value is an indicator of thylakoid dissociation. The structure of thylakoids is related to PSII function (Kirchhoff et al., 2007). Accordingly, the 4× samples had improved function of the thylakoids after 0.5% CO_2_ treatment. In addition,

The equation ϕ_po_ = Fv/Fm is widely used to reflect the maximum potential efficiency of PSII or the intrinsic quantum efficiency of the PSII units (Kitajima & Butler, 1975). As a multiparametric expression of the three main functional steps of photosynthetic activity by a PSII reaction center complex, the overall photosynthetic performance index (PI_ABS_) is also suitable to distinguish the photosynthetic performance of plants under various environmental conditions (Lepeduš et al., 2012). As shown in Figure 3, when exposed to 0.5% CO_2_, the 2× and 4× seedlings all exhibited decreases in Fv/Fm, indicating that 0.5% CO_2_ affected the behavior of PSII. At the same time, the different Fv/Fm ratio with 0.5% CO_2_ suggested that the photochemical reaction in 4× seedlings was different from that of 2× seedlings. Furthermore, the results of this study clearly reveal that the 4× species maintained a higher level of PI_ABS_ than did the 2× species under 0.5% CO_2_ conditions. Therefore, the photosynthetic system was damaged more severely in the 2× than in the 4× plants by 0.5% CO_2_ treatment.

As a consequence of the inhibition of PSII, excess light energy will accordingly lead to photoinhibition and oxidative stress, which can lead to a burst of reactive oxygen species (ROS, such as H_2_O_2_, ·OH, and O_2_^·−^) or lipid peroxidation (Asada, 1999; Yin, Pang, & Chen, 2009). Furthermore, an increase in ROS will result in chl loss and a decrease in photosynthetic CO_2_ assimilation (Ahmed et al., 2013). As evidence of ROS and lipid peroxidation generation, significantly increased H_2_O_2_ and MDA were observed in the 2× seedlings after 0.5% CO_2_ treatment (Figure 1). Additionally, Pn and chl contents were decreased in the 2× plants by 0.5% CO_2_ treatment (Figure 1, Tables 1 and 2). These observations confirmed that more serious oxidative stress occurred in the 2× than in the 4× plants after 0.5% CO_2_ treatment; that is, the 4× species had less oxidative stress.

To alleviate the damage initiated by oxidative stress, plants can modulate antioxidative enzymes such as SOD, POD, GR, etc. (Smirnoff and Wheeler 2000). The SOD, APX, and GR activities in leaves increase when exposed to elevated CO_2_ (Guo, Zhou, & Zhang, 2015). In our experiments, SOD, GR, and POD activities were decreased in the chloroplasts from the 2× leaves under the 0.5% CO_2_ condition (Figure 4). In contrast, the activities of POD and APX of the 4× leaves were remarkably stimulated by 0.5% CO_2_ (Figure 4). This result suggested that high CO_2_ stimulated an increase in the antioxidant enzyme activities of the 4× leaves to alleviate oxidative damage and indicted that the 4× species has a highly efficient defense system under elevated CO_2_ conditions to some extent. Although SOD, GR, and POD activities showed different changes between diploid and tetraploid black locust under specially elevated CO_2_ (0.5%), some sensitive enzymes have not been identified in the photosynthetic carbon metabolism. For instance, Umeton et al. (2012) found that 11 sensitive enzymes are confirmed to play a key role in terms of maximal CO_2_ uptake and minimal nitrogen consumption. Thus, in the future, these parameters or molecular methods should be used to gain a systematic and comprehensive understanding of polyploidy plants under elevated CO_2._

In some studies, increased numbers of osmiophilic globules in the cytoplasm and nucleolus‐associated bodies as well as electron dense material in vacuoles were observed in cadmium tolerant cells (Gzyl, Przymusiński, & Gwóźdź, 2009). And Bréhélin also found that the tocopherols stored in osmiophilic globules would be delivered to thylakoid membranes to scavenge ROS under oxidative stress (Bréhélin, Kessler, & van Wijk, 2007). In *Tillandsia albida (Bromeliaceae),* Papini found an increase in plastoglobules in plastids involved in autophagy (Papini, Mosti, & van Doorn, 2014). And Sallas et al. also reported that a non significant decrease in the number of plastoglobules in response to elevated CO_2_ in Norway spruce while an increase in number of chloroplast plastoglobules in Scots pine (Sallas, Luomala, Utriainen, Kainulainen, & Holopainen, 2003). It indicated that the response of plastoglobules depends on the plant species.

## CONFLICT OF INTERESTS

None declared.

## AUTHOR CONTRIBUTIONs

Fanjuan Meng and Yuan Cao conceived and designed the experiments; Yuan Cao and Mingquan Jiang performed the experiments; Shuo Liu, Mingquan Jiang, and Fuling Xu analyzed the data; Yuan Cao and Mingquan Jiang wrote the manuscript; Shuo Liu provided editorial advice.
